# Supplementary material for: Single-cell imaging reveals efficient nutrient uptake and growth of microalgae darkening the Greenland Ice Sheet
Source: Nat Commun. 2025 Feb 19;16:1521. doi: 10.1038/s41467-025-56664-6 (PMC11840010; doi:10.1038/s41467-025-56664-6)
Supplement: Supplementary file 1 — Supplementary Information [file 41467_2025_56664_MOESM1_ESM.pdf]

Supplementary Information for

Single-cell imaging reveals efficient nutrient uptake and growth of microalgae darkening the Greenland Ice Sheet

by Halbach et al.

Content:

Supplementary Figures 1-6
Supplementary Notes 1-2
Supplementary Tables 1-3
Supplementary References

Supplementary Figures

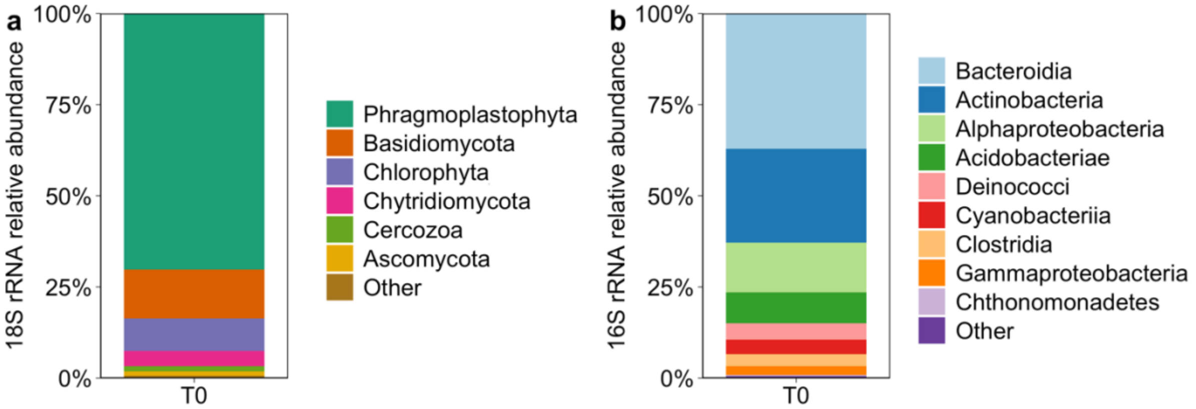

Supplementary Figure 1. Supraglacial community composition (T0) based on rRNA gene amplicon sequencing. (a) Eukaryotic community composition at the phylum level based on 18S rRNA gene relative abundance (n=1). Note that the phylum Phragmoplastophyta was comprised solely of *Mesotaeniaceae*, to which glacier ice algae belong. (b) Bacterial community composition at the class level based on 16S rRNA gene relative abundance (n=1). The amplicon sequencing data are available under BioProject ID PRJNA1209368 for the 16S data, and under BioProject ID PRJNA1209915 for the 18S data.

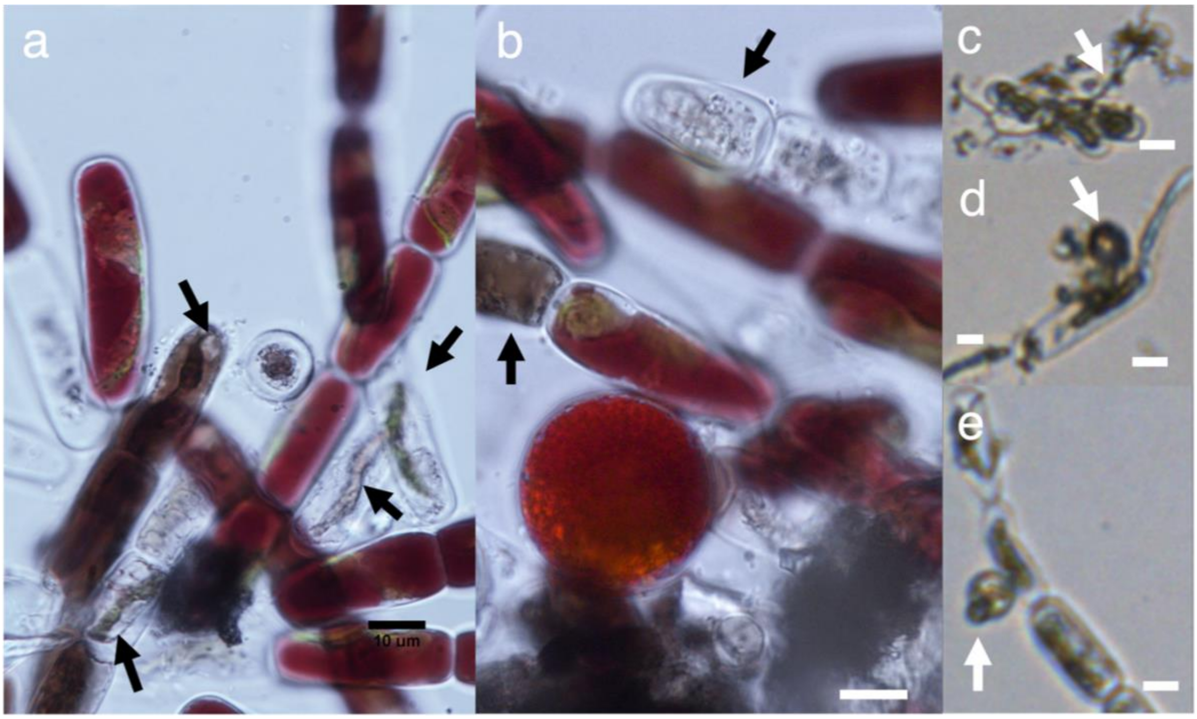

Supplementary Figure 2. Microscopic images of the glacial community at the start of the experiment (T0). (a, b) Microscopic images of unfixed samples, illustrating the phenotypic heterogeneity of *Ancylonema nordenskiöldii*. Black arrows indicate cells that are presumptively dead, exhibiting loss of pigmentation, or cells showing signs of degradation or infection. (c-e) FlowCam images of fixed samples, revealing infection of *A. nordenskiöldii* cells by chytrid fungi. Scale bars are 10 µm.

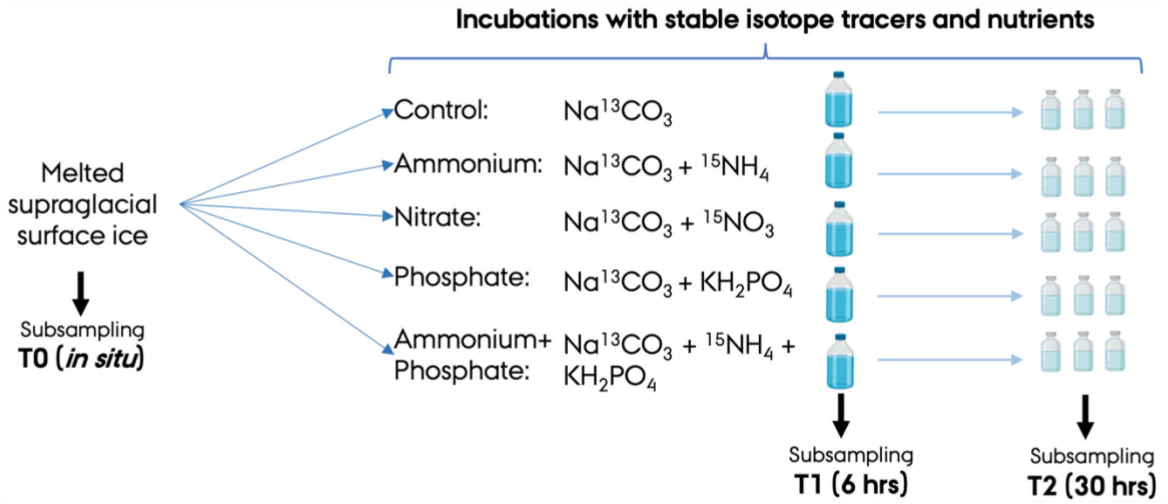

Supplementary Figure 3. Overview of samples used in this study: unmanipulated melted surface ice was subsampled, representing the "*in situ*" conditions and the T0 time point of the incubation experiment. Stable isotope tracers and nutrients were added to this melted glacier ice and incubated. The incubations were subsampled at T1 (after 6 hrs) from the blue cap bottles (1 only) and at T2 (after 30 hrs) from serum bottles (3 replicates each). Created in BioRender. Halbach, L. (2025) https://BioRender.com/w09s303.

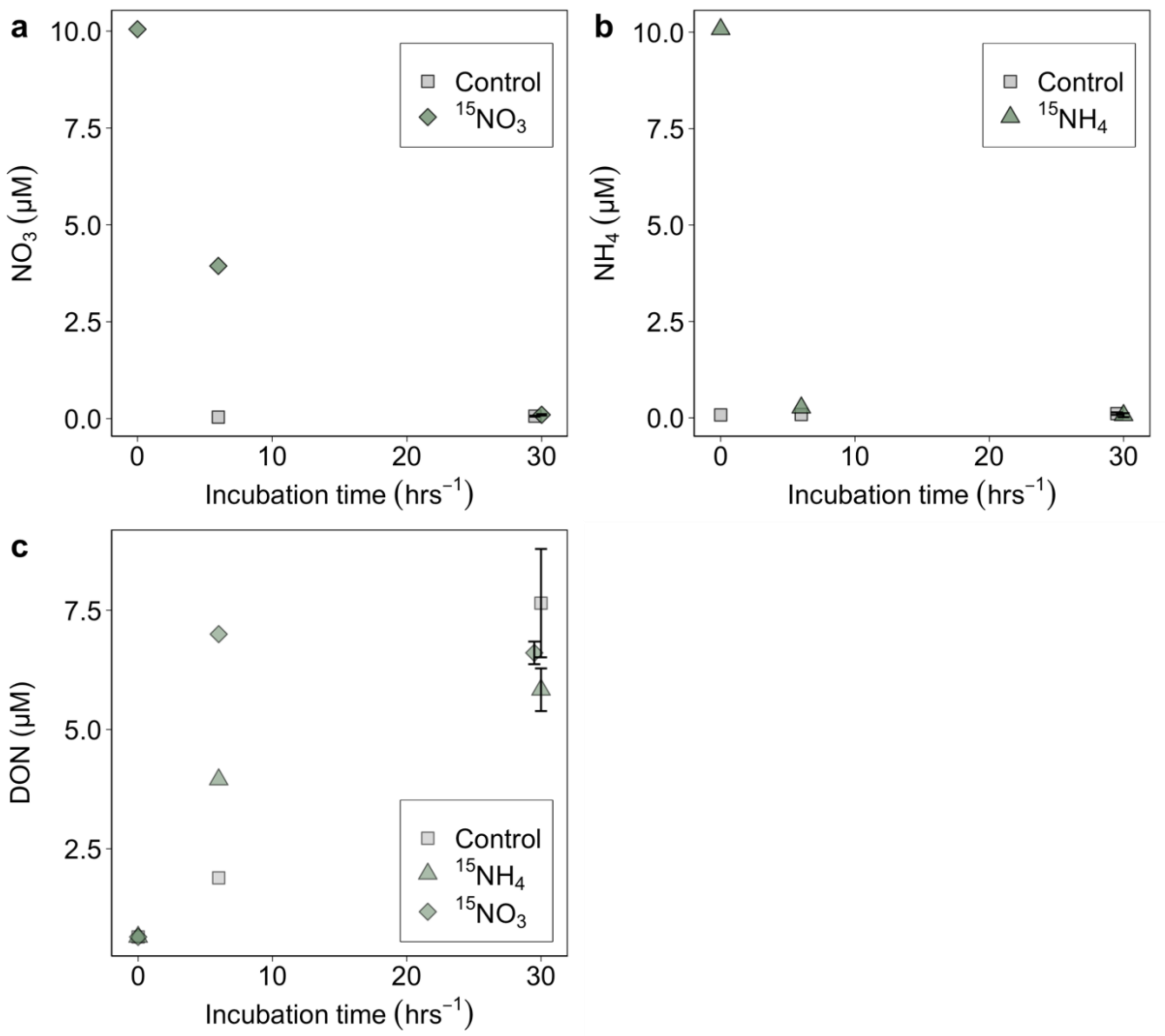

Supplementary Figure 4. Changes in NO_3, NH_4 and dissolved organic nitrogen (DON) concentrations during incubation (T0 with n=1 per treatment, T1: 6 hrs, with n=1 per treatment and T2: 30 hrs with n=3 per treatment). (a) NO_3^- in the ^{15}N-NO_3^- amended treatment and the control. (b) NH_4 in the ^{15}N-NH_4 amended treatment and the control. (c) DON concentrations in the control and ^{15}N-NH_4 and ^{15}N-NO_3 treatments. At T2 (n=3), the mean is plotted with error bars represent the range of the standard error.

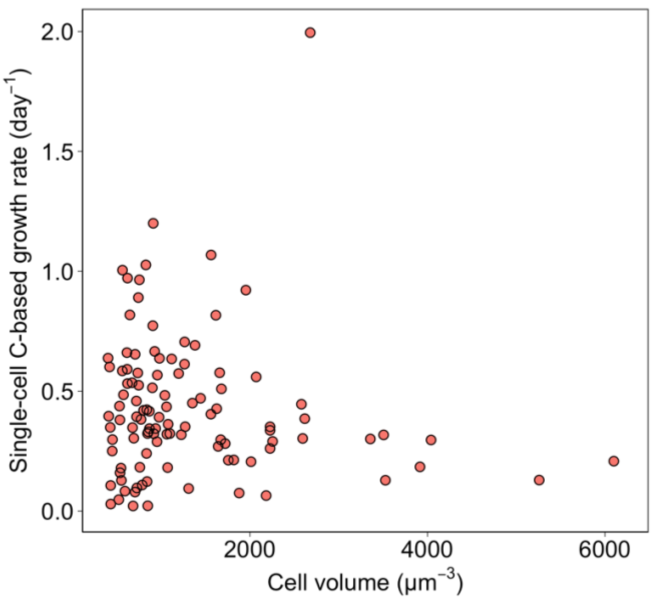

Supplementary Figure 5. Correlation between the cell specific C-based growth rates from the HR-SIMS measurements and cell volume of glacier ice algae from the Control, and NO_3^- and NH_4^+ treatments (n=125). Source data are provided as a Source Data file.

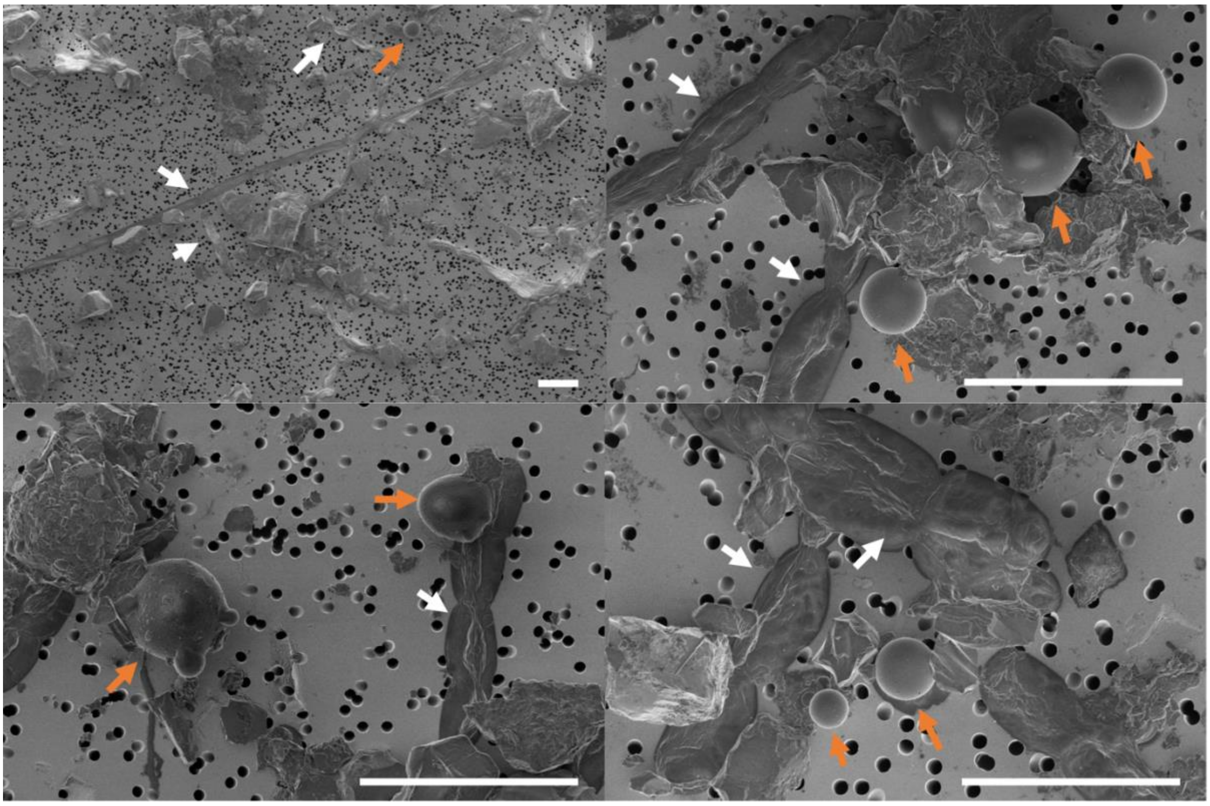

Supplementary Figure 6. SEM-images of the bulk POM after the incubations, fixed and dried on 3 µm pore size filters. Images show glacier ice algae (white arrows) along with other organisms, likely mainly snow algal cysts (orange arrows), inorganic particles and organic matter attached to the filter surface, cells and particles. Scale bars are 50 µm.

Supplementary Notes

Supplementary Note 1: ^{13}C-DIC labelling percentage

We determined the DIC concentrations of ^{13}C-DIC and ^{12}C-DIC in the melted glacier ice of T1 and T2 of the incubation experiment using GC-IRMS. In the control treatment, the total DIC (^{12}C-DIC+^{13}C-DIC) concentration measured at T1 was 283 µM. The ^{13}C-DIC concentration was 8 µM, thus implying that the DIC concentration of the melted glacier ice used for the experiment was 275 µM. This DIC concentration is much higher than the 15 µM previously measured in melted surface ice in Yallop et al.[1] or 44-70 µM in supraglacial streams in Andrews et al.[2]. This could be due to, to net heterotrophic activity and/or photooxidation. Carbonates were not present in the sample. Simple PHREEQ[3] modelling shows the pH, the partial pressure of dissolved CO_2 and the conductivity of the solution (Supplementary Table 6).

Supplementary Note 2: Uncertainty propagation

The uncertainties (σ) of the relative contribution of glacier ice algae to the bulk C and N uptake (Supplementary Tables 2 and 3) were determined by propagating the individual standard deviations associated with cell abundance (N_{cell}), single-cell assimilation rates ($assimilation_{cell}$) and bulk rates ($assimilation_{bulk}$) for the timepoints T1 and T2, respectively.

-$\sigma_{N\ cell}$; determined by the standard deviation of duplicate cell counts at T0 (n=2); or the standard deviation of the corrected algal abundance based on the biomass variation between the individual bottles (n=3 at T2, see description below)

-$\sigma_{assimilation\ cell}$; determined by the standard deviation of the single-cell measurements for the specific timepoint (for DIC at T1: n=11 and at T2: n=37; for NH_4^+ at T1: n=19 and at T2: n=26)

-$\sigma_{assimilation\ bulk}$; determined by the standard deviation of the bulk C or N uptake measurements for each individual timepoint (T1: n=1; T2: n=3)

Based on equation 4 in the main manuscript, the uncertainties in the relative contribution of glacier ice algae ($\sigma_{Contr\ cell}$) to bulk C and N uptake were calculated following the laws of error propagation for the different timepoints as follows:

$$\sigma_{Contr\ cell} = \overline{Contr\ cell} \times \sqrt{\left(\frac{\partial_{\text{N cell}}}{\overline{N\ cell}}\right)^2 + \left(\frac{\partial_{\text{assimilation cell}}}{\overline{assimilation\ cell}}\right)^2 + \left(\frac{\partial_{assimilation\ bulk}}{\overline{assimilation\ bulk}}\right)^2}$$

Additionally, to account for potential differences in algal abundance between bottles and timepoints, we calculated the contribution by the glacier ice algal population to bulk C and N uptake by correcting the algal abundance based on the fractional change in measured POC concentrations between T0 and T1, as well as between T0 and T2 (Supplementary Table 4). Variable biomass distributions between the bottles could be a consequence of (i) the rapid sinking out of paticles and/or (ii) heterogenous biomass distribution on the filter used for the bulk measurements – even despite carefully and thoroughly homogenising the melted glacier ice during the set-up of the experiment and prior to samplings. Using the corrected potential change in glacier ice algal abundance, we found that the estimated contribution of glacier ice algae to the bulk C and N uptake remained low (7-12% for C from DIC and 3-4% for N from NH_4^+; Supplementary Table 2 and 3).

Supplementary Tables

Supplementary Table 1. Overview of HR-SIMS-based measurements of single glacier ice algal cells. Estimates are presented as means with standard deviations.

Treatments	Sampling time	Incubation time (hrs)	n (number of cells)	C-based growth (day^{-1})			N-based growth (day^{-1})			C-assimilation (pmol C $cell^{-1}$ day^{-1})			N-assimilation (fmol N $cell^{-1}$ day^{-1})		
**Active population**															
Control	T1	6	11	0.66	±	0.37				9.72	±	5.45			
Control	T2	30	37	0.47	±	0.24				4.64	±	3.46			
NH_4^+	T1	6	19	0.19	±	0.11	0.18	±	0.05	4.31	±	4.20	183	±	137
NH_4^+	T2	30	26	0.40	±	0.37	0.07	±	0.03	4.27	±	6.97	37.7	±	23.2
NH_4^++PO_4^{3-}	T2	30	39	0.23	±	0.16	0.07	±	0.02	4.86	±	2.93	86.3	±	23.9
NO_3^-	T2	30	32	0.41	±	0.24	0.07	±	0.03	5.31	±	4.31	63.5	±	64.1
PO_4^{3-}	T2	30	56	0.20	±	0.11				4.38	±	2.25			
**Whole population (including nonactive cells)**															
Control	T1	6	15	0.48	±	0.44				7.06	±	6.49			
Control	T2	30	45	0.38	±	0.28				3.81	±	3.61			
NH_4^+	T1	6	20	0.18	±	0.11	0.17	±	0.06	4.09	±	4.20	177	±	136
NH_4^+	T2	30	28	0.37	±	0.37	0.07	±	0.03	3.97	±	6.80	35.1	±	24.2
NH_4^++PO_4^{3-}	T2	30	41	0.22	±	0.16	0.07	±	0.02	4.63	±	3.04	82.4	±	29.1
NO_3^-	T2	30	39	0.33	±	0.27	0.06	±	0.04	4.36	±	4.40	52.5	±	62.6
PO_4^{3-}	T2	30	56	0.20	±	0.11				4.38	±	2.25			

Supplementary Table 2. Relative contribution by glacier ice algal DIC assimilation to POM-based C uptake (from ^{13}C-DIC). Values are also presented in Table 2 in the main manuscript and are given as means ± standard deviation. Single-cell rates correspond to the rates of the active algal population fraction. The algal abundance at T0 was corrected for number of active cells within the population; i.e. 90% of the total cells, $1.46x10^{7}$ ± 0.11 cells L^{-1}). Bulk and single-cell rates are derived from the same replicate bottle for T1; for T2, single cell rates are derived from one replicate bottle, bulk rates from triplicate bottles. See Supplementary Note 2 for further details.

Parameter	GA abundance active		Single cell DIC assimilation GA	Total GA C assimilation (from ^{13}C-DIC)	Bulk C uptake (from ^{13}C-DIC)	**Est. contr. of GA to bulk C uptake**
Unit	(cells L^{-1})		(pmol C $cell^{-1}$ day^{-1})	(µg C L^{-1} day^{-1})	(µg C L^{-1} day^{-1})	**(%)**
T1	T0 cell abundance	$1.46\pm 0.1x10^{7}$	$9.7\pm5.5_{T1}$	$1700\pm970_{T1}$	22540_{T1}	**8±4 for T1**
	Est. T1 abundance*	$2.37\pm0.18x10^{7}$	$9.7\pm5.5_{T1}$	2770±1570	22540_{T1}	**12±7 for T1**
T2	T0 cell abundance	$1.46\pm 0.11x10^{7}$	$4.6\pm3.5_{T2}$	$820\pm610_{T2}$	$5380\pm1210_{T2}$	**15±12 for T2**
	Est. T2 abundance*	$0.68\pm0.19x10^{7}$	$4.6\pm3.5_{T2}$	$380\pm310_{T2}$	$5380\pm1210_{T2}$	**7±6 for T2**

*based on the fractional change in POC concentrations between T0 and T1, as well as, T0 and T2.

Supplementary Table 3. Relative contribution by glacier ice algal (GA) NH_4^+ assimilation to POM-based N uptake (from ^{15}N-NH_4^+). Values are also presented in Table 2 in the main manuscript and are given as means ± standard deviation. Single-cell rates correspond to the rates of the active/alive algal population fraction. The algal abundance at T0 was corrected for number of active cells within the population; i.e. 90% of the total cells, $1.46x10^{7}$ ± 0.11 cells L^{-1}). Bulk and single-cell rates are derived from the same replicate bottle for T1; For T2, single cell rates are derived from one replicate bottle, bulk rates from triplicate bottles. See Supplementary Note 2 for further details.

Parameter	GA abundance active		Single cell NH_4^+ assimilation GA	Total GA NH_4^+ assimilation (from ^{15}N-NH_4^+)	Bulk N uptake (from ^{15}N-NH_4^+)	**Est. contr. of GA to bulk N uptake**
Unit	(cells L^{-1})		(fmol N $cell^{-1}$ day^{-1})	(µg N L^{-1} day^{-1})	(µg N L^{-1} day^{-1})	**(%)**
T1	T0 cell abundance	$1.46\pm 0.11x10^{7}$	$183\pm137_{T1}$	$37\pm28_{T1}$	450_{T1}	**8±6 for T1**
	Est. T1 abundance*	$0.76\pm0.06x10^{7}$	$183\pm137_{T1}$	$19\pm15_{T1}$	450_{T1}	**4±3 for T1**
T2	T0 cell counts	$1.46\pm 0.11x10^{7}$	$38\pm23_{T2}$	$7.7\pm4.8_{T2}$	$127\pm14_{T2}$	**6±4 for T2**
	Est. T2 abundance*	$0.84\pm0.06x10^{7}$	$38\pm23_{T2}$	$4\pm2.7_{T2}$	$127\pm14_{T2}$	**3±2 for T2**

*based on the fractional change in POC concentration between T0 and T1, as well as, T0 and T2.

Supplementary Table 4. Variation in POC concentrations in the Control and $^{15}N-NH_4^+$ treatments between the timepoints, used to estimate the algal abundance (Supplementary Note 2).

	POC in Control (μmol C mL^{-1})		POC in $^{15}N-NH_4^+$ treatment (μmol C mL^{-1})	
T0	2.9		2.9	
T1	4.6	0.5±0.1x of T0 POC	1.5	0.5 x of T0 POC
T2	1.3±0.38	0.5x of T0 POC	1.5±0.11	0.6±0.04 x of T0 POC

Supplementary Table 5. Elemental composition of glacier ice algal cells given in atom% and mass%, derived by SEM-EDS.

	C	O	N	Na	S	Si	P	Al	Mg	K	Ca	Cl
mean (atom%)	78.8	14.4	4.2	1.2	1.0	0.3	0.2	0.1	<0.05	<0.05	<0.05	<0.05
SD	1.4	1.3	0.6	0.4	0.2	0.2	0.0	0.1				
mean (mass%)	71.5	17.3	4.4	2.0	2.4	0.5	0.4	0.1	<0.05	0.1	0.1	<0.05
SD	6.1	1.9	0.8	0.6	0.5	0.4	0.1	0.1		<0.05	0.1	
n	48	48	48	48	48	48	48	48	48	48	48	48

Supplementary Table 6. Chemical parameters to characterise the melted glacier ice prior to the start of the incubation experiments (T0) and after 6 hrs of the incubation experiment (T1). * denotes calculated parameters.

**Parameter**	**Value**	**Unit**	**Sampling time**
DIC	275	μM	T1
pH	5.18-5.62		T0
Conductivity	4.6	μS/cm	T0
$\log_{10} p(CO_2)$*	-2.47	atms	calculated

Supplementary References

1. Yallop, M. L. *et al.* Photophysiology and albedo-changing potential of the ice algal community on the surface of the Greenland ice sheet. *ISME J* **6**, 2302–2313 (2012).
2. Andrews, M. G., Jacobson, A. D., Osburn, M. R. & Flynn, T. M. Dissolved Carbon Dynamics in Meltwaters From the Russell Glacier, Greenland Ice Sheet. *J Geophys Res Biogeosci* **123**, 2922–2940 (2018).
3. Parkhurst, D. L. & Appelo, C. A. J. Description of input and examples for PHREEQC version 3 - A computer program for speciation, batch-reaction, one-dimensional transport, and inverse geochemical calculations. in *U.S. Geological Survey Techniques and Methods, book 6,* 497 (2013).
